# Repetitive Transcranial Magnetic Stimulation (rTMS) an an augmentation strategy in a sample of patients with treatment-resistant depression: a comparison of traditional and accelerated protocols

**DOI:** 10.1192/j.eurpsy.2025.2121

**Published:** 2025-08-26

**Authors:** D. Conti, C. Bucca, N. Girone, L. Larini, M. Vismara, B. Benatti, B. Dell’Osso

**Affiliations:** 1 Department of Mental Health, Department of Biomedical and Clinical Sciences Luigi Sacco; 2 ”Aldo Ravelli” Center for Neurotechnology and Brain Therapeutic, University of Milan, Milan, Italy; 3 Department of Psychiatry and Behavioral Sciences, Bipolar Disorders Clinic, Stanford University, Stanford, United States

## Abstract

**Introduction:**

Repetitive transcranial magnetic stimulation (rTMS) is a clinically validated treatment, included in international guidelines for managing treatment-resistant depression (TRD). The conventional daily administration of rTMS protocols over several weeks can pose challenges for patients, such as work interruptions and commuting difficulties. To enhance the antidepressant effects and minimize the duration of treatment, increasing the frequency of daily rTMS sessions has been proposed as a potentially more effective approach.

**Objectives:**

This study aims to compare the efficacy and tolerability of standard versus accelerated rTMS protocols as supplementary treatments for patients with TRD

**Methods:**

24 patients diagnosed with major depressive episodes (either unipolar or bipolar) and classified as partial responders or non-responders to adequate pharmacological treatment were randomized into two groups. One group received standard rTMS (one session daily, five days a week, over four weeks; n=9), while the other underwent accelerated rTMS (two sessions daily, five days a week, over two weeks; n=15). Both groups were treated on the left dorsolateral prefrontal cortex with high-frequency stimulation (10 Hz) at 120% of the motor threshold, delivering 3000 pulses per session. Primary outcome measures included scores from the Hamilton Depression Rating Scale (HAM-D), Montgomery-Åsberg Depression Rating Scale (MADRS), and Clinical Global Impressions-Severity (CGI-S) at baseline (T0), post-treatment (T1), and during follow-ups at one month (T2) and three months (T3). Tolerability was assessed based on reported adverse effects. Paired Samples t-Test was employed for continuous variable comparisons, while t-Test was used to analyze differences between groups.

**Results:**

Analysis of the overall sample revealed a significant reduction in HAM-D, MADRS, and CGI-S scores from T0 to T1 (p<0.001). These improvements were maintained at the one-month and three-month follow-ups (T1 vs T2: p=0.726, p=1.00, p=0.803; T2 vs T3: p=0.105, p=0.594, p=0.653). No significant differences were observed in response and remission rates between the two protocols (T1: p=0.722; T2: p=0.727; T3: p=0.979). The only reported side effect was mild, transient headaches during stimulation, with a minimal dropout rate (0.25%).

**Conclusions:**

These preliminary findings align with previous literature, suggesting similar efficacy and tolerability between accelerated and standard rTMS protocols. Future studies with larger, controlled, and blinded designs are warranted to validate these results and explore treatment parameters for accelerated rTMS.

**Disclosure of Interest:**

None Declared

